# Coracoclavicular Double-Button Fixation of Displaced Lateral Clavicular Fracture in a Patient without Coracoid Process

**DOI:** 10.1155/2019/6506951

**Published:** 2019-12-23

**Authors:** Rodrigo Nicolás Brandariz, Maria Guillermina Bruchmann, Franco Luis De Cicco, Luciano Andres Rossi, Ignacio Tanoira, Maximiliano Ranalletta

**Affiliations:** Department of Trauma and Orthopaedics, “Carlos E. Ottolenghi” Institute, Hospital Italiano de Buenos Aires, Juan D. Perón 4190 (C1181ACH), Buenos Aires, Argentina

## Abstract

**Background:**

Displaced distal clavicular fractures are generally operated on because of the high nonunion rate after nonoperative treatment. Several surgical techniques have been developed to reduce the nonunion rate and improve functional outcomes. One of them is closed reduction and minimally invasive coracoclavicular double-button fixation, which requires the integrity of the coracoid process to be performed.

**Case Summary:**

We present a 35-year-old male patient who had been successfully subjected to a modified Latarjet procedure for glenohumeral instability with bony defect treatment, and 7 months later suffered a distal clavicle fracture in his ipsilateral shoulder. With a CT scan, we analyzed the coracoid remnant size (7 mm), and we consider it enough to perform a minimally invasive double-button technique, using this remnant as a distal fixation.

**Postoperative and Follow-Up:**

Radiographic and clinical fracture union occurred 10 weeks after the procedure. The patient returned to sports at the same level he had before surgery and achieved full strength and range of motion.

**Conclusion:**

Closed reduction and minimally invasive double-button fixation of displaced distal clavicular fractures is a safe, reproducible, and versatile technique, which can even be performed without an intact coracoid process.

## 1. Introduction

Approximately 10-15% percent of clavicle fractures occur in the distal third fragment [1–3].

Healing complications of distal clavicular fractures are well known, with nonunion rates as high as 44% and delayed union in up to 75% of the fractures [1, 4]. In the subgroup of young active patients, nonunion is frequently associated with persistent pain, restriction of movement, and loss of strength and endurance of the shoulder [5]. Therefore, in young active patients with a displaced distal third clavicle fracture, surgical treatment is generally indicated [6].

Multiple surgical techniques have been proposed to manage distal clavicular fractures including the use of a tension band, the modified Weaver-Dunn procedure, and coracoclavicular screw fixation or locking plates [1, 7]. None of these techniques have been universally accepted, and each one has its own complications, including wire migration, symptomatic hardware [8], osteolysis of the acromion [9], and migration of the hook into the acromion [10].

Recently, open and arthroscopic techniques using button fixation have been proposed for the treatment of these fractures, in an attempt to decrease the complications associated with other procedures [11–16]. The key condition to perform this procedure is the integrity of the coracoid process so that the lower button can be toggled on the inferior cortex of the coracoid process. However, in a patient who has undergone a Latarjet procedure, stable fixation in the distal button could be jeopardized.

We present the case of a young athlete who underwent a previous Latarjet procedure for shoulder instability and 7 months later suffered a distal clavicle fracture in his ipsilateral limb, stabilized with an arthroscopic double-button fixation set in place in the remnant base of the coracoid process.

To our knowledge, there are no previous publications describing this association.

## 2. Case Presentation

A 35-year-old male was injured in his right shoulder in a motorcycle accident. After the accident, he was unable to lift his arm so he came to our hospital. As relevant information, seven months earlier, he had undergone a successful Latarjet procedure for the treatment of anterior shoulder instability. By the time he had the accident, he had resumed his normal daily activities and returned to sports at the same level he played prior to the surgical procedure. No complications were reported. Healing of the coracoid graft was confirmed in a CT scan at 4 months follow-up postoperatively.

**Figure 1:**
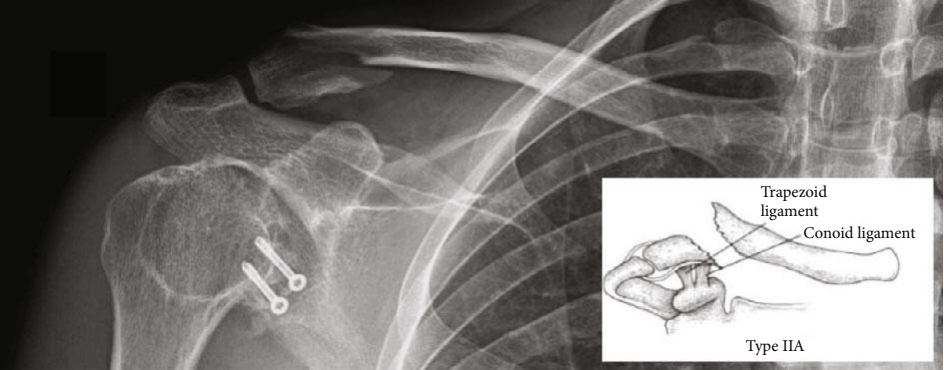
Standard radiograph of the right clavicle. It shows distal clavicle fracture classified as a Neer type IIA fracture pattern. Screws from the Latarjet procedure are in a good position without signs of loosening. The remnant of the coracoid process is also shown.

Physical examination denoted deformity, tenderness, and swelling on his right shoulder. The range of motion was limited, flexion was 80°, and abduction was about 45°, and a constant score was made which amounted to 34 points. Neurovascular examination was normal. Standard radiographs of the shoulder and right clavicle were made as well as a Zanca projection. They revealed a distal clavicle fracture classified as a Neer type IIA fracture pattern. Screws from the Latarjet procedure were in a good position without signs of loosening. They also showed the remnant of the coracoid process, according to surgical history (Figure 1).

**Figure 2:**
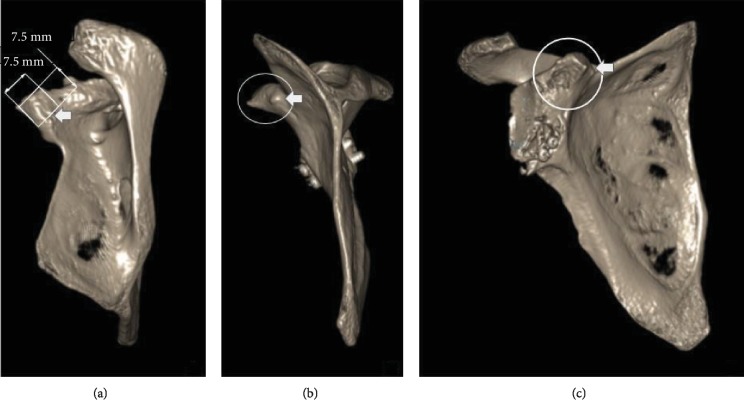
CT scan showing the coracoid process remnant's measure. (a) Scapula superior view, measure from the base to the distal end of the previous osteotomy; total length and width was 7.5 mm. (b) Scapula medial view. (c) Scapula anterior view.

Due to this background, a CT scan was performed in order to measure the coracoid process remnant which would affect the decision on the surgical technique chosen. We measured it from the base to the distal end of the previous osteotomy (Figure 2). Total length was 7.5 mm, and it was decided to use a double-button fixation system, since the drilling for the placement of the device has a width of 4 mm, leaving enough bone available for the stabilization of the button.

Surgery was performed with an interscalene nerve block and the patient placed in the beach-chair position without traction. Standard antibiotic prophylaxis was used (cefazoline, 2 grams intravenously). The ipsilateral arm and shoulder were prepared and draped in a sterile fashion. First, a posterior portal and a diagnostic arthroscopy were performed. The coracoid bone graft of the Latarjet procedure previously performed was found in the correct position and healed. No associated injuries were found. An anterior working portal was then created through direct vision through the rotator interval. The rotator interval was opened and the subscapularis bursa and scar tissue were resected until the remnant of the base of the coracoid process was visualized and carefully cleaned using a radiofrequency ablator. An anterolateral accessory portal was created and the arthroscope was changed using a switch stick to have a lateral vision. The coracoid remnant was measured arthroscopically confirming the length of 7.5 mm previously analyzed in the CT scan.

**Figure 3:**
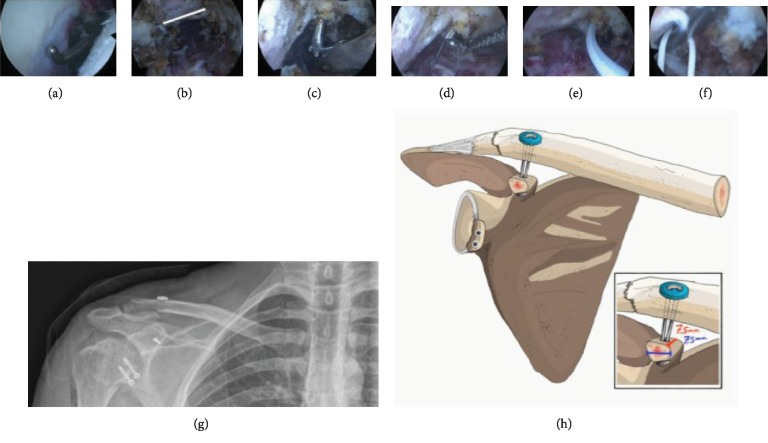
Arthroscopic views and postoperative radiography. (a) Coracoid bone graft of the previous Latarjet procedure found in the correct position and healed. (b) Remnant of the base of the coracoid process carefully cleaned using a radiofrequency ablator. (c) Drill introduced in the anterior portal and centered on the under face of the remnant of the base of the coracoid process. (d) The FLOOP system (South American Implants) inserted through the tunnels using the wire. (e, f) The button flipped under the surface of the coracoid process. (g) Postoperative radiography confirms an accurate reduction in the fracture and position of the buttons. (h) Schematic view of surgical planning. (Image contributed by De Cicco FL, MD.)

A drill guide was then introduced in the anterior portal and centered on the under face of the remnant of the base of the coracoid process. Good positioning and support of the guide in the coracoid remnant were observed. Then, a 3 cm incision was made over the clavicle, and the guide was placed down and positioned 15 mm medial to the fracture in its center. A 2 mm Kirschner wire was inserted and a 4 mm hole was created with a cannulated drill through the clavicle and the remnant of the coracoid process. A guidewire was passed through the drill and retrieved through the anterior portal. The FLOOP system (South American Implants) was then inserted through the tunnels using the wire, and the button was flipped under the surface of the coracoid process. After the flipping of the button, the clavicle button was placed and the system was tightened through its blocking capacity when adjusting, until anatomic clavicle alignment was achieved. The button was then secured with 5 knots. This allowed reduction of the proximal part of the fracture. Fluoroscopy was performed to confirm an accurate reduction in the fracture and position of the buttons. The patient was discharged the same day after surgery with our usual standard of antibiotic prophylaxis (500 milligrams of cephalexin every 6 hours for two days), without antithrombotic prophylaxis (Figure 3).

**Figure 4:**
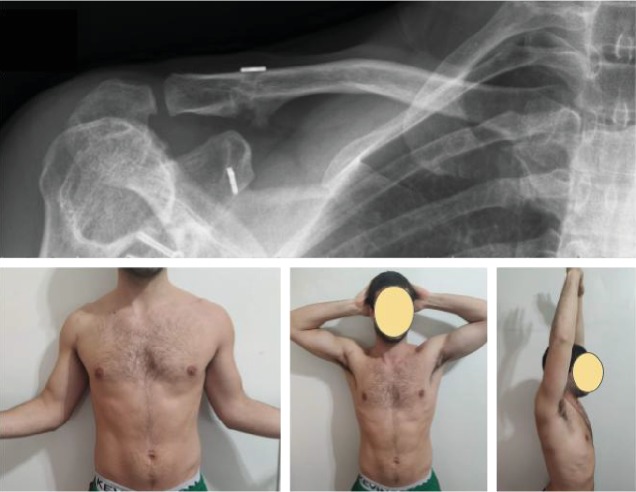
10 weeks after the procedure, radiographic union, and clinical assay. Full shoulder range of motion had been achieved, and shoulder strength was near 100%.

Postoperative, the arm was supported with a sling for 4 weeks. The patient was not permitted to elevate the arm above 90° in any plane during the first 3 weeks. The patient was allowed to bear weight on his arm from the sixth week onwards. After week 8, full shoulder active range of motion in all planes was allowed, with an increase in the intensity of strength and functional training for a gradual return to activities and sports. The patient was allowed to return to sports when radiographic and clinical fracture union had occurred (10 weeks after procedure), full shoulder range of motion had been achieved, and shoulder strength was near 100%. (Figure 4). Clinical follow-up was conducted weekly during the first month and then monthly until fracture healing was achieved. The radiological evaluation consisted of a frontal clavicle radiograph and Zanca projection. One year after surgery, the patient indicated no pain on a visual analogue scale and the constant score was 95 points (the preoperative score was 34 points). The patient had no symptoms or signs referable to the acromioclavicular joint on specific testing.

## 3. Discussion

Displaced distal clavicular fractures are generally operated on because of the high nonunion rate after nonoperative treatment [3, 9].

Several surgical techniques have been described including hook plates, locking plates, coracoclavicular screw fixation, and double-button fixation [7]. However, there is no consensus regarding which method is better regarding consolidation, function, and complications.

In 2018, Boonard et al. [7] conducted a systematic review and meta-analysis to compare shoulder function and complications between different fixation methods for distal clavicle fracture in 600 patients. The authors reported that locking plates associated with better functional results and lower complication rates.

However, in fractures compromising the coracoclavicular ligaments, or with small lateral fragments (Neer IIa or b), the treatment with locking plates may be insufficient (due to the associated ligament injury) or technically unfeasible (due to lateral bone stock) [17].

Recently, several studies reported that arthroscopically assisted or open coracoclavicular stabilization using a suture button device for unstable lateral clavicle fractures showed satisfactory fracture stability with low complication rates [16, 18].

This has also been proven in biomechanical studies, where coracoclavicular button had more than 75% greater strength than the traditional locking plate alone [19].

Regarding the surgical technique, it is essential to assure the correct placement of the lower button at the base of the coracoid process with the double-button system. Ferreira et al. performed a biomechanical study and described that it is critical to place the tunnels leaving enough peripheral bone stock to avoid complications [20].

In fact, among the most frequent failures with this type of fixation are the fractures of the coracoid due to poor positioning of the tunnels [21].

Generally, these fractures occur in the medial or lateral cortices, because the length of the intact coracoid process doubles its width [20].

In this case, we performed a preoperative CT scan to accurately measure the remnant coracoid process and analyze whether the bone was sufficient for the button fixation. Total length was 7.5 mm. In the previous surgery, we obtained the coracoid bone graft by performing an osteotomy at a position just anterior to the insertion of the coracoclavicular ligaments at the coracoid base; the measure we obtained made us suspect that this osteotomy was performed anteriorly than usual.

Descriptions in revision anterior cruciate ligament reconstruction set a limit of 3 mm bone bridge between tunnels to ensure a safe fixation in the lateral femoral condyle [22].

Having this concept in mind, and knowing the drilling of the tunnel has a diameter of 4 mm, the minimum length necessary for the coracoid remnant to support the button has to be 7 mm, leaving 3 mm of intact bone. Measures performed in the CT scan showed that the length of 7.5 mm would be sufficient to perform this technique.

One year postoperatively, the patient has excellent functional outcomes and radiographic consolidation without loosening or any other complications.

In conclusion, closed reduction and minimally invasive double-button fixation of displaced lateral clavicular fractures is a safe, reproducible, and versatile technique, which can even be performed without an intact coracoid process. We recommend a minimum length of 7 mm of the coracoid remnant to perform the procedure safely.
